# Pregnancy and Emerging Diseases

**DOI:** 10.3201/eid1303.061345

**Published:** 2007-03

**Authors:** Martha Anker

**Affiliations:** * University of Massachusetts, Amherst, Massachusetts, USA

**Keywords:** Severe acute respiratory syndrome, pregnancy, emerging diseases, outbreaks, letter

## To the Editor

The November 2006 issue of Emerging Infectious Diseases featured 2 perspectives (*1*,*2*) that highlighted the need for strategies to prevent and treat pregnant patients during outbreaks of new or emerging diseases or during bioterrorist attacks. However, neither article discussed implications for a surveillance strategy.

Based on my previous experience at the World Health Organization (WHO) with the severe acute respiratory syndrome (SARS) outbreak, I propose several practical steps for such a strategy: l) systematic identification and reporting of cases in pregnant women, 2) estimation of the number of cases in pregnant women, 3) international clinical networks to share treatment and infection control experience, and 4) standard protocols for sharing clinical and treatment information between nations.

First, cases in pregnant patients should be systematically identified and reported during outbreaks, by including information on pregnancy status and duration of the pregnancy in case-report forms for new diseases. This is important for several reasons. First, case-patients that come to attention in an ad hoc fashion may provide a biased view of outcome, since those with a poor outcome are more likely to draw attention. Second, although pregnancy is not rare, the number of cases in pregnant women in outbreaks of new or emerging diseases in any 1 location may be too small for meaningful analysis.

Unfortunately, during the SARS epidemic, pregnancy status was not included on the international case-reporting forms. Although some countries systematically tested for and recorded pregnancy status, other countries did not. As a result, valuable information was lost, and outcomes for pregnant women could not be properly assessed.

A rough estimate can be made of the number of pregnant women in a particular country likely to have a particular disease such as SARS. Assuming equal attack rates for pregnant and nonpregnant women, the number of pregnant women having a disease can be estimated as equal to three fourths of the sum over 5-year age groups of the product of the number of female patients in the 5-year age group by the age-specific fertility rate for that age group.^1^^,^^2^^,^^3^ While the assumption of equal attack rates does not hold for all infectious diseases, it is a good starting point for a new disease about which there is little information.

Using this method for SARS resulted in an estimate of 119 cases in pregnant women (Table). For most countries, the estimated number of pregnant case-patients was reasonably close to the total number of pregnant case-patients that could be identified in the scientific literature through web searches (column 3) supplemented by cases identified through informal sources, such as emails and at WHO meetings and conference calls (column 4). China was an exception; 84 pregnant case-patients were estimated for China, but only 5 case-patients were identified, all from the same hospital in China.

**Table:** Pregnant patients with severe acute respiratory syndrome (SARS), by country

Country	Estimated no. pregnant SARS patients*	No. pregnant SARS patients reported in scientific literature†	No. SARS cases identified through informal sources‡
China	84§	5	0
Taiwan	6	0	2
Hong Kong	17	12	0
Singapore	5	0	4
Vietnam	1	0	1
Canada	4§	1	0
All other	2§	2¶	0
Total	119	20	7

*National estimate equal 3/4 of the sum over all 5-y age groups of the product of the number of female patients in the 5-y age group by the age-specific fertility rate for that age group. †Cases were found by author through intensive scientific literature search. ‡Identified through World Health Organization meetings, conference calls and emails. §The country-specific age-sex distribution of cases was not available for this country; the combined age-sex distribution from all available patients from other countries was used in this estimate. For this reason estimate for this country may be less precise. ¶Both patients were from the United States.

These estimates have limitations. They do not consider subnational differences in fertility, such as differences for specific ethnic or occupational groups, or rural-urban differences. Nonetheless, they provide a ballpark figure that can be used to assess the extent of pregnancy-related cases.

The estimates were useful during the SARS outbreak in raising awareness of the issues surrounding pregnancy and SARS. As a result of such awareness, WHO formed a clinical network to share clinical experiences regarding treatment of pregnant patients as well as experiences with infection control during obstetrical procedures. Although this network was established rather late during the SARS outbreak, it did result in useful interchanges between nationals in different countries during conference calls.

The network discussed the establishment of a standardized database for sharing detailed clinical information on the course, outcome, and treatment of pregnant SARS patients. However, this database was never created. Although, fortunately, the scientific literature contains summaries of experiences with 20 case-patients, this is not a substitute for a systematic and reasonably complete database of experiences. To be sure that such a database is established for any new disease, protocols for data sharing should be prepared in advance, and all possible administrative barriers to sharing information should be addressed.

In conclusion, pregnant women are an important group at high risk for outbreaks of new diseases. This situation requires appropriate strategies for surveillance. I have identified several measures that I believe could be used in this regard.

## Footnotes

1 It is necessary to multiply by 3/4, because live births take 9 months, on average.

2 The estimated number of pregnant women is based on expected number of live births, and the estimate does not take into consideration fetal loss, premature delivery, or multiple births.

3 Estimates can be made by using an electronic spreadsheet. Age-specific fertility rates can be drawn from United Nations Population Division data available for all countries.
