# Supplementary material for: Pregnancy and Emerging Diseases
Source: Emerg Infect Dis. 2007 Mar;13(3):518–9. doi: 10.3201/eid1303.061345 (PMC2725895; doi:10.3201/eid1303.061345)
Supplement: Related texts

# Reply: In Response

**Authors:** Denise J. Jamieson, Sonja A. Rasmussen, Janet D. Cragan, Joanne Cono

**Affiliations:** * Centers for Disease Control and Prevention, Atlanta, Georgia, USA

We thank Ms. Anker for describing some of the surveillance challenges during the severe acute respiratory syndrome (SARS) pandemic and for supporting the idea that during an infectious disease outbreak or public health emergency, pregnant women are a population deserving special consideration. We also understand and appreciate the challenges she describes in systematically identifying and reporting pregnancies during a public health emergency response such as occurred with SARS. In the aftermath of Hurricane Katrina, we have recognized that it is critical that pregnancy information be collected systematically as part of the medical intake process. This can be accomplished by asking a simple, short question about pregnancy status (i.e.,“Are you or do you think you could be pregnant?”) as women are being evaluated and processed as part of an emergency response plan. Urine pregnancy testing may be used to verify pregnancy status for women who are unsure (*2*). Besides helping tailor the provision of healthcare in these settings, pregnancy status information should also be used to track pregnancy outcomes.

We agree with Ms. Anker regarding the importance of pregnancy surveillance efforts. In June 2003 the Centers for Disease Control and Prevention (CDC) announced the first evidence of community-acquired monkeypox in the United States. Because this outbreak was linked to infected prairie dogs often kept as pets by small children, exposed household members included mothers, some of whom were pregnant. Unfortunately, although outbreaks of monkeypox in Africa had previously been well described, these descriptions did not include much information on the natural history of monkeypox among pregnant women (*3*). This example highlights the importance of ensuring that pregnancy information is systematically collected to plan and respond to future outbreaks.

In addition to surveillance of pregnant women in a known outbreak or emergency response, pregnant women should be included in efforts to detect novel disease threats. Unusual patterns of disease among pregnant women may be an early sign of emerging disease in the general population. In their role as frontline physicians, obstetrician-gynecologists and other healthcare providers who care for pregnant women may be among the first to encounter patients with novel infectious disease threats (*4*).

Finally, with Ms. Anker, we support coordinated international efforts to collect and monitor the pregnancy status of affected women during an outbreak or emergency response and are working on ways at CDC to prepare for future threats.

## References

[1] Anker M. Pregnancy and emerging diseases [letter]. Emerg Infect Dis. 2007;13:518.1755212410.3201/eid1303.061345PMC2725895

[2] Callaghan W, Rasmussen SA, Jamieson DJ, Ventura SJ, Farr SL, Sutton PD, Health concerns of women and infants in times of natural disasters: lessons learned from Hurricane Katrina. Maternal Child Health. 2007 Jan 25; [Epub ahead of print]10.1007/s10995-007-0177-417253147

[3] Jamieson DJ, Cono J, Richards CL, Treadwell TA. The role of the obstetrician-gynecologist in emerging infectious diseases: monkeypox and pregnancy. Obstet Gynecol. 2004;103:754–6.1505156910.1097/01.AOG.0000114987.76424.6d

[4] Jamieson DJ, Ellis JE, Jernigan DB, Treadwell TA. Emerging infectious disease outbreaks: old lessons and new challenges for obstetrician-gynecologists. Am J Obstet Gynecol. 2006;194:1546–55. 10.1016/j.ajog.2005.06.06216731070PMC7093849
